# CBCT assessment of radicular volume loss after rapid maxillary expansion: A systematic review

**DOI:** 10.4317/jced.54745

**Published:** 2018-05-01

**Authors:** Antonino Lo Giudice, Cosimo Galletti, Cosme Gay-Escoda, Rosalia Leonardi

**Affiliations:** 1 Department of Medical-Surgical Specialties – Section of Orthodontics, School of Dentistry, University of Catania, Azienda Ospedaliero - Universitaria “PoliclinicoVittorio Emanuele”, Via S. Sofia, 78 - 95123 Catania, Italy; 2 Department of Biomedical and Dental Sciences and Morphofunctional Imaging – Section of Orthodontics, School of Dentistry, University of Messina, Policlinico Universitario “G. Martino”, Via Consolare Valeria - 98123 Messina, Italy; 3 Oral and Maxillo-facial Surgery Department. School of Dentistry, University of Barcelona. Campus de Bellvitge UB, L’Hospitalet de Llobregat, Barcelona, Spain

## Abstract

**Background:**

The present systematic review analyzed the current literature to investigate whether rapid maxillary expansion (RME) causes radicular resorption, assessed by cone-beam computed tomography (CBCT).

**Material and Methods:**

Eighteen electronic databases and reference lists of studies were searched up to November 2017. Grey literature was also screened. To be included, articles must be human studies on growing subjects with transversal maxillary deficiency treated with maxillary expansion protocol and with 3-D radiographic assessment of radicular volume by CBCT images. Two authors independently performed study selection, data extraction, and risk of bias assessment. Study characteristics (study design, sample size, age, sex, skeletal maturity, type of appliance, daily activation, teeth evaluated, CBCT settings), and study outcomes (radicular volume loss) were reported according to the PRISMA statement.

**Results:**

Only 3 articles were considered eligible and an individual analysis of the selected articles was undertaken. The risk of bias assessment revealed low methodological quality for all the studies included. In all the considered studies, significant radicular volume loss was observed in posterior teeth, following RME. When reported in percentage, the radicular volumetric loss was similar between anchored (first molars and first premolars) and unanchored teeth (second premolars).

**Conclusions:**

A preliminary evaluation of the patient-related risk factors for RR is warmly advisable when administering RME.

** Key words:**RME, maxillary expansion, root resorption, external root resorption.

## Introduction

Root resorption (RR) represents a physiologic or pathologic condition resulting in the dissolution of cementum and dentin of dental roots (1). It represents an inevitable side effect of the orthodontic treatment with individual predisposition and orthodontic mechanics playing a role as etiological factors (2). However, the implication of RR in terms of viability and physiologic function of the involved teeth is still unclear (3).

Rapid maxillary expansion (RME) is the standard treatment for the correction of transversal maxillary deficiency (4,5) in growing subjects. During the active phase, heavy forces are transmitted to the maxilla by the anchored teeth. Those forces determine the hyalinization of periodontal ligament, preventing dental movement (6). Later, with the permanence of residual forces stored in the appliance, the orthodontic effect initiates and the sequelae of periodontal ligament hyalinization may occur, i.e., buccal bone plate and RR (7,8).

Nevertheless, the diagnosis of RR is difficult due to the lack of clinical pathognomonic symptoms. During orthodontic treatment, indeed, clinicians may notice RR through routine examinations such as panoramic or periapical radiographs (9). However, conventional 2-D radiography presents large inaccuracies in detecting RR, especially when it is located at the buccal or lingual root surface (10) as it may occur after RME.

Within the last few years, cone-beam computed tomography (CBCT) has been widely used in dentistry. It provides 3-D images of dental structures without projection errors and with less artifacts compared to conventional CT (11). CBCT has greater accuracy in detecting in-vitro simulated resorption cavities compared to conventional periapical radiographs (9). Furthermore, CBCT allows detecting radicular volume changes (12). Since RR is a kind of dental volume loss, the assessment of radicular volume is superior compared to a bidimensional analysis of root length (12).

In the present study, we systematically reviewed, for the first time, the bibliographic data from studies investigating RR following RME therapy by means of CBCT radiography in young patients.

## Material and Methods

The present systematic review is consistent with the guidelines of the Cochrane Handbook for Systematic Reviews of Interventions (version 5.1.0) and is reported according to the PRISMA statement (13-15).

Two authors (A.L.G. and C.G.) independently carried out the selection of the studies, data collection and the assessment of risk of bias. Any disagreement was resolved by discussion with a third author (C.G.E.). This study was registered in the National Institute of Health Research database with an appropriate protocol number (http://www.crd.york.ac.uk/PROSPERO Protocol: CRD42016052064).

-Search Method

Searches were conducted on several electronic databases to find out articles concerning the effects of no-surgical skeletal maxillary expansion on dental root resorption, published up November 2017. Specific electronic databases were also searched for conference abstracts, dissertations, conference proceedings and unpublished literature (grey literature). The strategy search was adjusted for each database and is reported in  Table 1,  Table 1 continue,  Table 1 continue-1. The reference lists of the articles eligible for inclusion were also manually reviewed. No restriction was applied to language, publication year, or status. Finally, authors were contacted to obtain specific data or info not provided in their article.

**Table 1:**
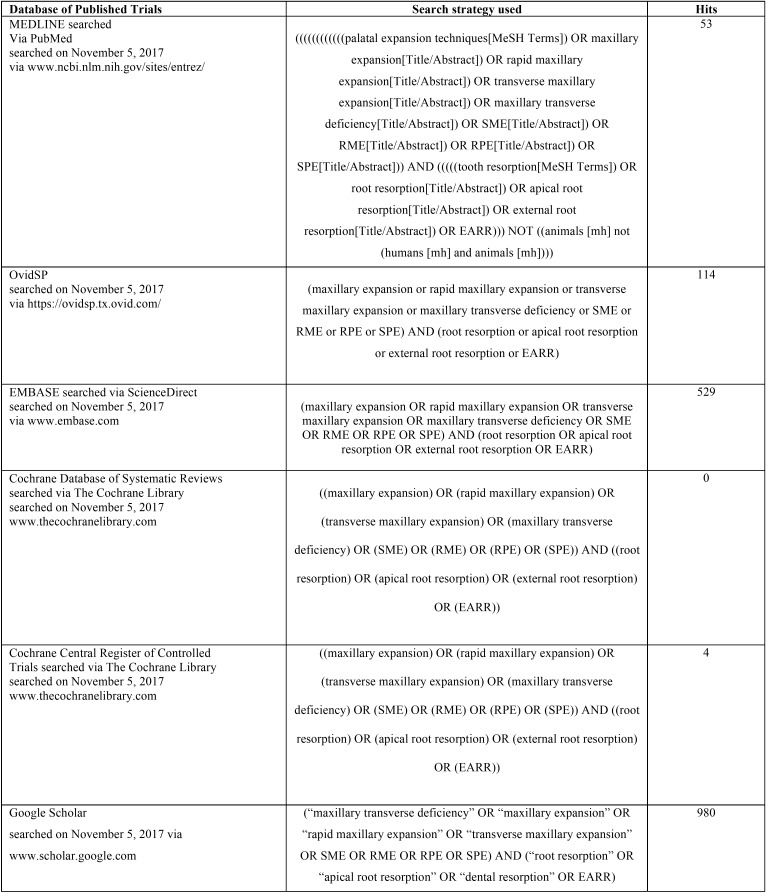
Strategy searches used for the eighteen electronic databases.

**Table 1 continue:**
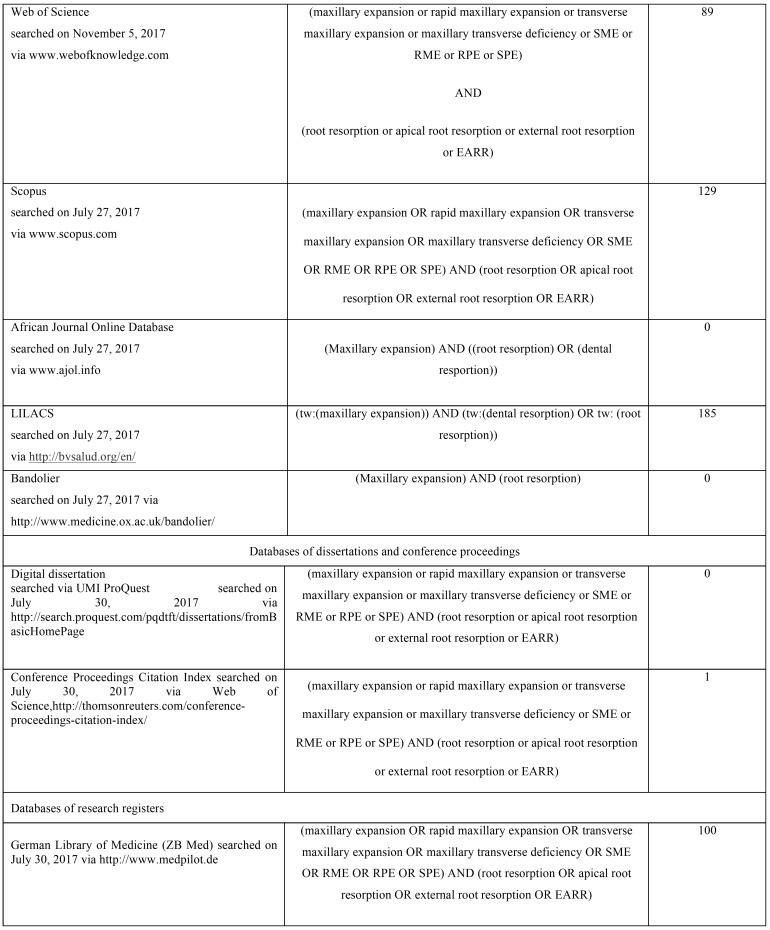
Strategy searches used for the eighteen electronic databases.

**Table 1 continue-1:**
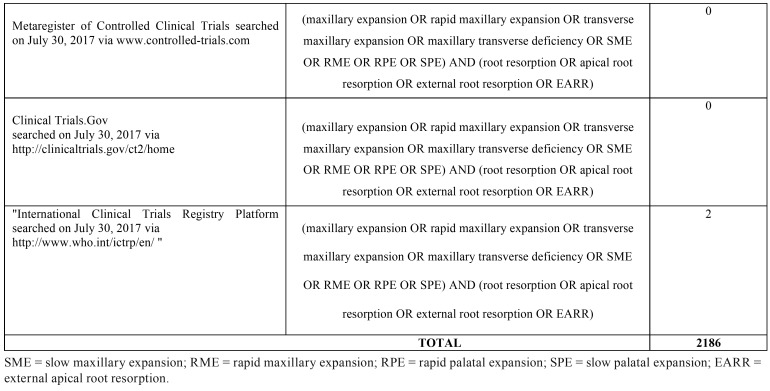
Strategy searches used for the eighteen electronic databases.

-Selection of studies

According to the PICOS (population, intervention, comparison, outcome, study design) format, the following inclusion criteria were selected to assess the eligibility of the studies: related human clinical studies; studies conducted on growing patients with maxillary transverse deficiency (Population); non-surgical skeletal maxillary expansion therapy (Intervention); control group represented by pre-treatment radicular volumetric measurements of posterior permanent teeth (Comparison) assessed by CBCT; post-treatment radicular volume loss of the same teeth (Outcomes measured); randomized or non-randomized clinical trials, cohort studies, case–control and retrospective studies (Study design). Studies including patients with previous orthodontic treatment, periodontal diseases, endodontic treatment of posterior teeth, tooth agenesis or with anomalies in form, shape, or structured and congenital syndromes were excluded.

In a first phase, the authors screened all titles and abstracts retrieved from the databases and selected the studies that assessed radiographically root resorption after maxillary expansion. The reference lists of the studies included in phase 1 were also screened to retrieve additional eligible articles. In a second phase, the same authors reviewed the full texts of the studies selected in phase 1 and considered eligible only those assessing volumetric root resorption by means of CBCT images. The level of agreement between the two reviewers was assessed by the Cohen’s kappa statistics.

-Data collection

Data extraction form was developed to collect the characteristics (study design, sample size, age, sex, skeletal maturity, type of appliance, daily activation, teeth evaluated, observation period, CBCT settings) and the outcomes (radicular volume loss) of the included studies. The Cohen’s kappa statistics was performed to assess the agreement between the two authors.

-Assessment of risk of bias

Risk of bias assessment was performed using the Downs and Black scale16 as suggested by the Cochrane Handbook for Systematic Reviews of Interventions (13-15) The instrument consisted of 27 questions evaluating (1) reporting [10 questions], (2) external validity [3 questions], (3) internal validity or bias [7 questions], (4) internal validity or confounding or selection bias [6 questions], and (5) power [1 question]. According to this scale, answers were scored from 0 to 1 point, except for 2 items: reporting domain (question number 5) scored from 0 to 2 points and power analysis (last question) scored from 0 to 5 points. Consequently, the total maximum score that a clinical trial could receive was 32 points. The level of agreement between the two review authors was assessed with the Cohen’s kappa statistic.

-Data analysis

Meta-analysis of the extracted data would have been considered only if the methodology was consistent among the included studies and if they reported equivalent volumetric measurements.

## Results and Discussion

-Selection of studies

2186 articles were initially identified and 1636 remained after duplicates’ removal; a total of 1629 articles were excluded after the reading of titles and abstracts and the full texts of the remained 7 articles were retrieved (Phase 1) (17-23). After reading the full texts of those studies, only 3 articles were considered eligible for the final inclusion in the present systematic review (Phase 2) (17-19).

**Figure 1:**
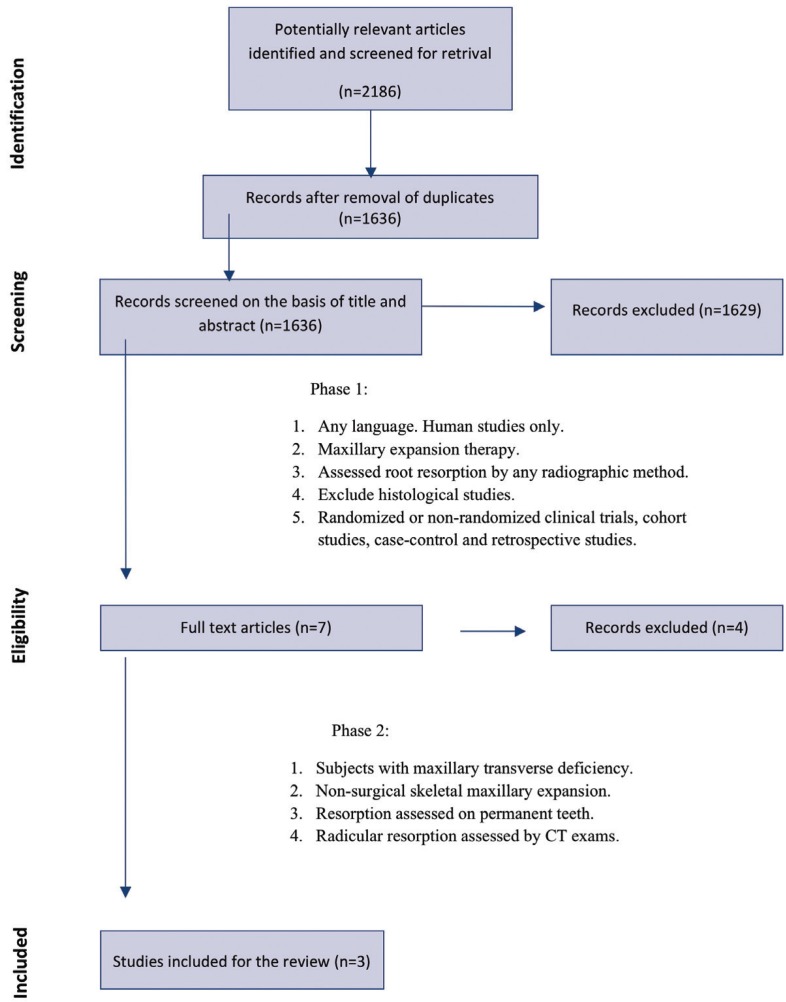
Flow chart of study selection performed according to the PRISMA guidelines.

Figure 1 shows the flow diagram for the selection of studies, and the excluded articles alongside with the reasons for their exclusions in both Phase 1 and Phase 2 are shown respectively in  Table 2 and  Table 3. The agreement between the reviewers was highly reliable, with a kappa value of 0.984.

**Table 2:**
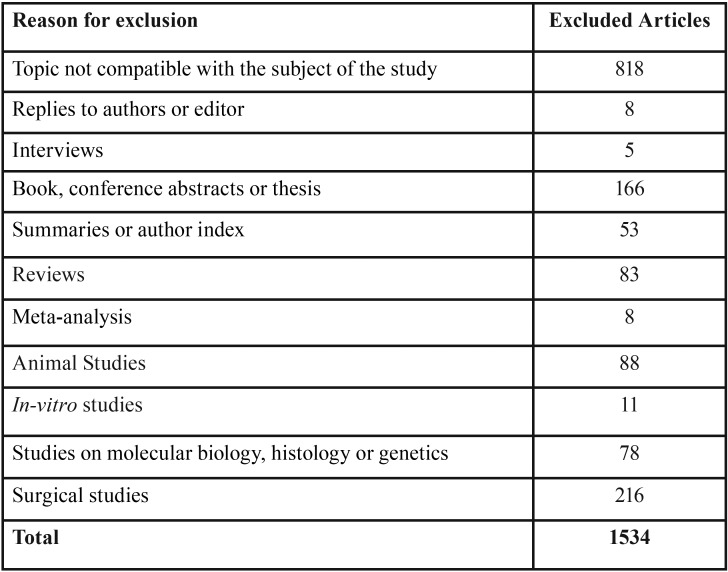
Excluded articles with reason for exclusion during phase 1 of studies selection.

**Table 3:**
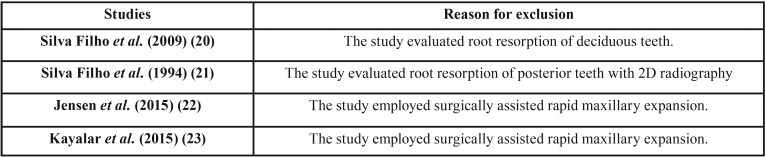
Excluded articles with reason for exclusion during phase 2 of studies selection.

-Study characteristics

The characteristics of the three studies included in this systematic review are summarized in  Table 4. Two studies reported a retrospective cohort (17,18) and one was designed as prospective study (19) All the selected articles investigated the radicular volume loss of posterior teeth after skeletal maxillary expansion by CBCT. All studies took place in a university setting and included both male and female participants. The mean age of the patients was almost identical in the three studies: 12.8 years (17,19) and 12.7 years (18). Only one study presented a control group and was constituted by the corresponding mandibular teeth (17). The following types of maxillary expander were used: Hyrax expander with bands on the first premolars and first molars (18,19). Hyrax expander with extended arms to the second and first premolars (17) and Haas expander with bands on the first premolars and first molars (19). The protocol of expansion ranged from 0.25 mm17 to 0.50 mm18,19 of daily activation. One study (19) reported data of skeletal maturity that were based on hand-wrist radiograph. The timing of radiological assessment was different among the three studies: immediately after the active treatment phase (18,19) and after 4.8 months of appliance retention; (17) moreover, one study (19) reported data of follow-up after six months of appliance retention. According to the design of the appliances tested, two investigations (18,19) assessed the radicular volume loss of first molars, first premolars (anchored teeth) and second premolars (unanchored teeth), whereas one study (17) analyzed the first molars (anchored teeth) and the first premolars (wire-supported teeth). All CBCT images were at high resolution with different exposure parameters and with a reported voxel size of 0.18 mm (17), 0.20 mm (19) and 0.30 mm (18). The methodology and the volumetric measurements were heterogeneous among the three investigations: one study (18) measured the volume of each root of first molars, while two studies (17,19) measured the absolute radicular volume. In two studies (17,18) the roots were separated over the cement-enamel junction due to the presence of bands on molar teeth, and one study (20) included the cervical region in the assessment of radicular volume. The agreement between reviewers was highly reliable with a kappa value of 0.997.

**Table 4:**
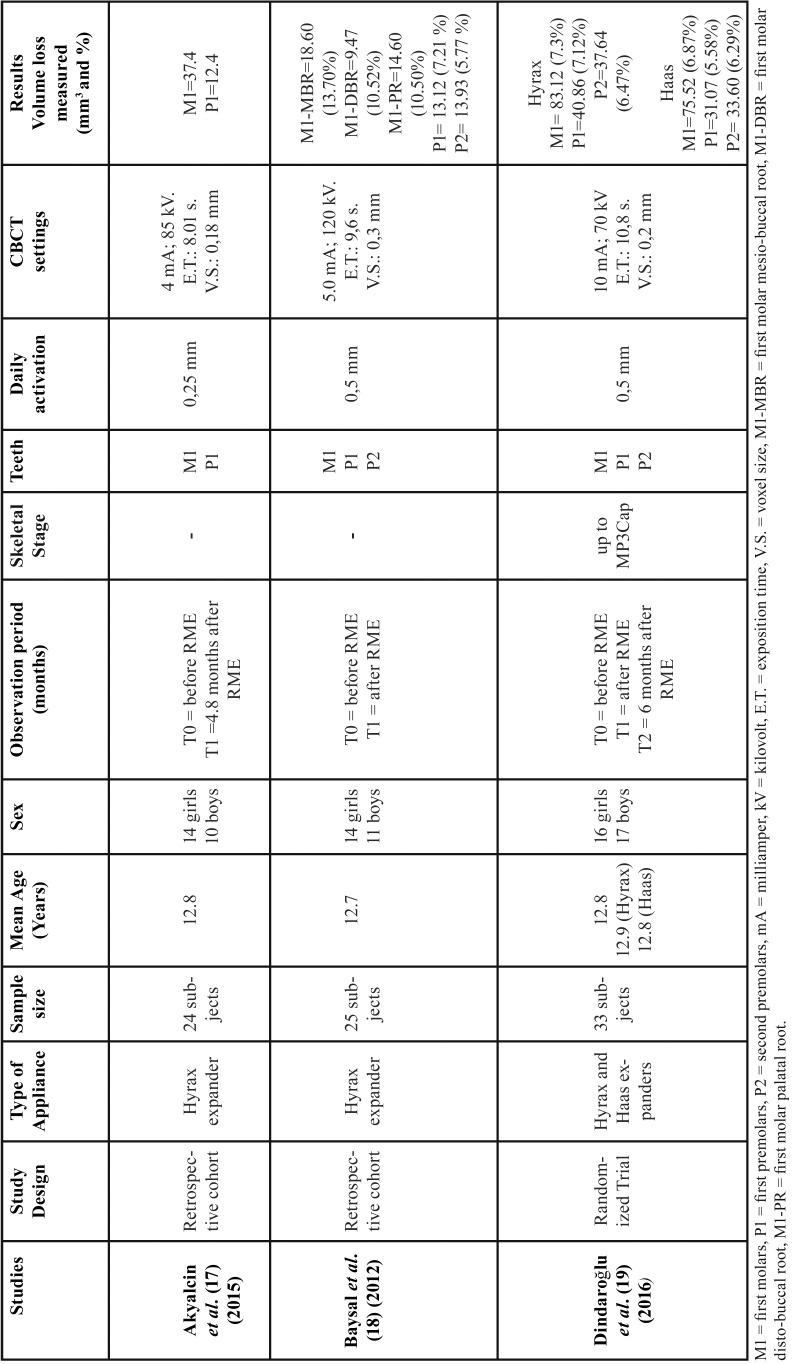
Characteristics of the studies considered for the final analysis.

-Assessment of risk of bias

The overall score for the assessment of the risk of bias for each included studies (17-19) was evaluated as low, indicating poor reporting and experimental design in accordance with to the Downs and Black scale ( Table 5) (16). The agreement between reviewers was highly reliable with a kappa value of 0.904.

**Table 5:**
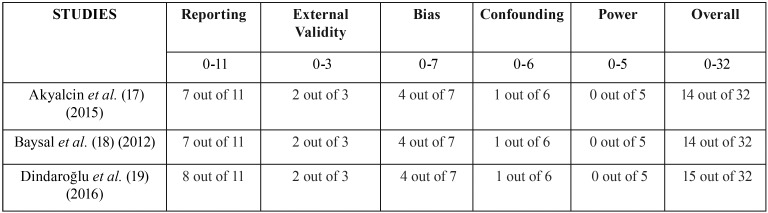
Risk of bias evaluation of selected studies.

-Data analysis

A meta-analysis was not feasible due to the heterogeneity in study designs and the absence of comparable volumetric measurements among the included studies. Consequently, the risk of bias across the studies could not be performed and a descriptive analysis of the reported results was conducted.

To the best of our knowledge, this is the first systematic review investigating the existing literature in order to evaluate whether RME therapy causes the RR of posterior teeth, as assessed *in vivo* by CBCT. A previous systematic review has dealt with 2-D radiographic assessment of RR following RME, (24) however the authors reported that the literature was inconclusive due to the limitations of periapical radiographs to reveal RR, except for frank apical resorption.

We performed a wide and accurate bibliographic search and we found only 3 articles eligible for inclusion. The methodological quality of the included studies was limited, as assessed by the employed bias assessment tool. Also, these studies failed to give the higher level of scientific evidence, which is only attainable through the use of randomized clinical trials (25). Further prospective 3D studies, with adequate control groups, different retention periods and follow-ups are required in order to quantitatively assess RR in patients undergoing RME therapy. However, this is hardly possible due to the recommendations of the British Orthodontic Society and the American Association of Orthodontists concerning the use of CBCT and the risks related to ionizing radiation exposure for each patient (26-29). In the absence of the highest level of evidence, clinicians have to make decisions based on lower levels of evidence such those provided by the study included in the present systematic review.

A meta-analysis was not possible because the methodology and the reported information were heterogeneous among the included studies. Hence, we reported a descriptive analysis of the obtained results.

According to the findings of this systematic review, skeletal maxillary expansion induces radicular volume loss of posterior teeth. By comparing radicular volumes before and after RME, all the included studies (17-19) consistently reported the first molars as the teeth mostly affected by radicular resorption with mean values of volume loss ranging from 83.12 mm3 to 37.4 mm3. First premolars showed radicular volume loss ranging from 40.86 mm3 to 13.12 mm3 and second premolars from 37.64 mm3 to 13.93 mm3 ( Table 4). Two studies (18,19) reported also the mean percentage of radicular volume loss: for the first molars, Dindaroğlu *et al.* (19) found a total mean volume loss of 6.87%, whereas Baysal *et al.* (18) reported the percentage of volume loss for each root i.e., 13.70% for the mesio-buccal root, 10.52% for the disto-buccal root and 10.50% for the palatal root. The mean percentage of volume loss ranged from 5.58% to 7.21% for the first premolars and from 5.77% to 6.47% for the second premolars. No statistically significant differences were found in the percentage of radicular volume loss among the teeth investigated (18,19).

The extension of radicular resorption after RME can be influenced by the retention period and by the timing of radiographic examination. In this respect, the cumulating forces stored in the appliance and the relapse forces may sustain the resorption process even during the retention phase (3,30). Meanwhile, the cementum reparative process starts when the orthodontic forces are below a certain level (31). Thus, both processes may occur simultaneously during the retention period. In such cases, the resorption process may prevail over the cementum repair to an extent that may depend on several variables including patient age, skeletal maturity, subjective biological response and the protocol of maxillary expansion. This makes difficult to detect the true extension of RR (24). Among the considered articles, only Dindaroğlu *et al.* (19) reported a follow-up evaluation of radicular volumes after RME. The authors found a small radicular volumetric recovery that ranged from 4.43 mm3 for first premolars in the Hyrax group to 25.14 mm3 for first molars in the Haas group. Although cementum repair can only be assessed by histological analysis of radicular structural changes (8), it can be deduced that the volumetric differences of each tooth, between T1 (after expansion) and T2 (6 months retention period), reflect the amount of reparative cementum.19 Such volumetric recovery may be underestimated because CBCT cannot detect the repaired cementum until it is completely re-mineralized (31). These findings, however, would suggest that 6 months of retention may be insufficient to appreciate the full extension of tissue repair.

Patients’ mean age was about 12.8 years in all the three studies (17-19). At these ages, the root development could not be fully completed, especially for second maxillary premolars. Thus, the volumetric data reported following RME therapy could be partially attributed to the disruption of roots’ development. In this respect, Akyalcin *et al.* (17) found a slight increase in both volume and radicular length in the control group (mandibular first molars and premolars) compared to the tested group. However, no conclusion can be drawn due to the statistical and clinical irrelevance of the reported data and because such increment in the control group could be attributed to the continuous formation of cementum as consequence of changes in the occlusion (17).

It has been hypothesized that tissue borne appliances caused less radicular resorption than tooth-borne appliances due to the acrylic plate that distribute the expansion forces to supporting tissue.10 In this respect, Dindaroğlu *et al.* (19) found that the radicular volume loss was greater in the Hyrax group compared to the Haas group. However, such differences were found to be not statistically significant (10.6 mm3 for first molars, 9.79 mm3 for first premolars, and 4.04 mm3 for second premolars) are clinically irrelevant.

Clinicians often use a modified version of the original Hyrax and Haas appliances featuring a wire-supported anchorage system instead of bands on first premolars. A recent histological study, with split-mouth design, reported no significant differences of root resorption between the first premolars supported by the two anchorage systems.32 According to these findings, Akyalcin *et al.*, (17) which employed a wire-supported anchorage of first premolars, reported significant radicular volume loss of these teeth (12.4 mm3). However, no comparison can be made with the findings of the two other studies, i.e. Baysal17 (13.12 mm3) and Dindaroğlu (19) (Hyrax group = 40.86 mm3; Haas group = 31.07 mm3), due to the heterogeneity in the methodology applied.

CBCT technology is reported to possess a good accuracy in assessing volumetric measurements of teeth and RR (12,33). However, such technology is affected by a systematic error consisting in the manual segmentation procedures that are influenced by voxel sizes, artifacts and quality of images (12,33). In this respect, Wang *et al.* (12) reported that CBCT with a 125-μm voxel size (0.125 mm) could identify cavities larger than 3.47 mm3 but failed to detect cavities smaller than 1.07 mm3 because of manual segmentation. In the studies included in this systematic review, the voxel resolutions were 0.30 mm (17) 0.20 mm (18) and 0.18 mm (17). Thus, it may be the case that small volumetric changes might not be discerned, determining their underestimation.

-Clinical implications

The implication of RR in terms of viability and physiologic function of the involved teeth is still unclear (25) Clinicians, however, are called to keep the risk of tissue damage as low as possible while performing therapeutic procedures. In this respect, we suggest to clinicians: 1) to perform an accurate anamnesis in order to identify the patient-related risk factors for root resorption (3,34) 2) in mixed dentition, to use primary first molars as anchored teeth if they are steady, 3) to avoid RME in youngster insofar as higher forces are applied to the dentition due to the difficulty in opening the mid-palatal suture (35). In this case, clinicians may refer to surgically assisted expansion procedures or orthognathic surgery for the treatment of transversal maxillary deficiency. Moreover, when posterior teeth are at higher risk of periodontal damage and the amount of maxillary transversal deficiency does not justify such invasive surgical procedures, a bone-born palatal could represent a valid alternative (36).

## Conclusions

• RME causes radicular volume loss of posterior teeth with the first molars as the teeth mostly affected by radicular resorption, as assessed by CBCT.

• When reported in percentage, the radicular volumetric loss was similar between anchored (first molars and first premolars) and unanchored teeth (second premolars).

• Clinicians must take into account such adverse effect when they choose to treat transversal maxillary deficiency using a palatal expander anchored to the permanent dentition.
